# Realistic Real-Time Outdoor Rendering in Augmented Reality

**DOI:** 10.1371/journal.pone.0108334

**Published:** 2014-09-30

**Authors:** Hoshang Kolivand, Mohd Shahrizal Sunar

**Affiliations:** MaGIC-X (Media and Games Innovation Centre of Excellence), UTM-IRDA Digital Media Centre, Universiti Teknologi Malaysia, Skudai, Johor, Malaysia; Xiamen University, China

## Abstract

Realistic rendering techniques of outdoor Augmented Reality (AR) has been an attractive topic since the last two decades considering the sizeable amount of publications in computer graphics. Realistic virtual objects in outdoor rendering AR systems require sophisticated effects such as: shadows, daylight and interactions between sky colours and virtual as well as real objects. A few realistic rendering techniques have been designed to overcome this obstacle, most of which are related to non real-time rendering. However, the problem still remains, especially in outdoor rendering. This paper proposed a much newer, unique technique to achieve realistic real-time outdoor rendering, while taking into account the interaction between sky colours and objects in AR systems with respect to shadows in any specific location, date and time. This approach involves three main phases, which cover different outdoor AR rendering requirements. Firstly, sky colour was generated with respect to the position of the sun. Second step involves the shadow generation algorithm, Z-Partitioning: Gaussian and Fog Shadow Maps (Z-GaF Shadow Maps). Lastly, a technique to integrate sky colours and shadows through its effects on virtual objects in the AR system, is introduced. The experimental results reveal that the proposed technique has significantly improved the realism of real-time outdoor AR rendering, thus solving the problem of realistic AR systems.

## Introduction

In contrast to indoor rendering, outdoor rendering consists of more components such, for example: position of the sun, sky colours, shadows, rainbows, haze, trees, grass and etc. This paper begins, attempting a working definition for some of the more important parameters for outdoor rendering. Position of the sun, sky colours, shadows and interaction between the sky colours and other objects are the more significant components when it comes to outdoor environments. These factors are taken into account because they are the prominent components of outdoor environments [1] [2].

Over the past two decades, Augmented Reality (AR) has become one of the most enthralling topics, not only in computer graphics but also in other fields [3] [4] [5], beckoning researchers on obtaining greater results. In AR, realism can be achieved through entering shadows as well as inducing interaction between objects [6] [7] [8] [9].

**Figure 1:**
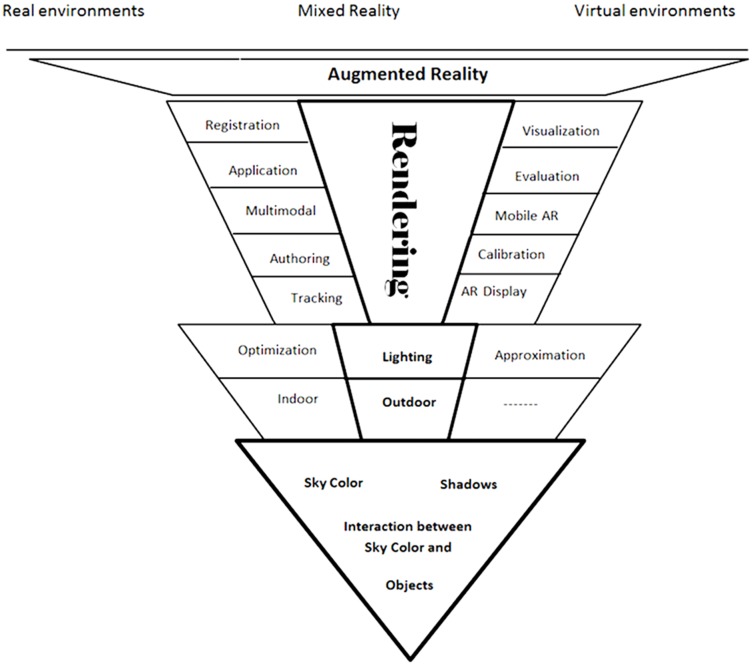
Research focus area.

In general, realistic augmented reality has been a critical point in computer graphics before the turn of 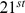 century [10]. Here, to produce a realistic virtual object in real outdoor environments, position of the sun, sky colours, shadows and interaction between sky colours and objects are taken into account. Figure 1 represents the research area. The final focus area is shown as well as all open issues in AR.

Studies concering sky colours and shadows are the main resources for outdoor components using grammars with sets of rules. Rendering outdoor components is studied for visualization of natural scenes in different contexts: animators, ecosystem simulations, video games, design architectures and flight simulators [11] [12].

Sky illumination on virtual objects is the most significant factor in outdoor rendering not only in virtual environments but also in augmented reality systems [8] [13] [14] [15] [16] [17] [18] [19] [20]. Generating sky colours as a background for each outdoor scene is an essential aspect to make it more realistic. Illustrations of the sky has become very crucial, as many buildings are designed, so that the sky or other surrounding scenes are emblazoned through the building windows [21].

Shadows are one of the prominent factors taken into consideration when it comes to enhancement of realistic outdoor environments; by realising the depth of the scene, using the distance between the objects present. Without shadows and shadow casters, it is strenuous to assimilate, as well as appreciate the real size of objects when compared to others, which are placed further away.

Semi-soft shadows are meant to be used in outdoor environments, considering their distance from the light source-sun is cosmic. Wider areas in outdoor environments require an altered and somewhat particular shadow generating technique to reveal: the difference between shadows of the objects that are located closer to the camera's point of view and of those, that are located further ahead.

Casting virtual shadows on other virtual objects and real environments should be supported in realistic outdoor environments, hence an advanced technique is introduced to achieve this. The presented shadow generating technique is easily implemented not only in any virtual environments but also in all AR systems.

In outdoor AR games, the designer must choose the colour of virtual objects, to create quality photo that reflects sky colour variations. The choice of the colour suitable for outdoor AR games, requires extensive investigation, even though accurate results have not been attained yet [22] [23] especially in the case of real-time [24] [7] [17] [8] [9]. Revealing the effects of sky colour on the virtual objects is the final objective taken into account to enhance the realism of the outdoor AR system.

An appropriate technique is in order to integrate all mentioned factors in augmented reality. The technique removes the problems associated with colour selection. Furthermore, it has the additional advantage of observing the interaction between sky colours and virtual objects like what can be seen on real objects during a day [25] [26] [27] [28].

This article includes two new ideas to generate a realistic real-time outdoor environment. A semi-soft shadow generating technique with high quality and lower cost of rendering is presented; as it is required for wide scale outdoor environments. Implementing the proposed shadows technique in AR systems is further contribution of this study to have virtual shadows on other virtual and real objects. The integration technique in an AR system can be expressed as additional achievements towards the main goal of this piece.

### Previous Works

Blinn [29] were the first researcher who used the indirect illumination to demonstrate the actual distance between objects which is known as: reflection mapping. The method is improved by [30] then [31]. They used diffuse and specular reflection to corresponding components of reflection. Nishita [32] and Ward [33] illuminated real-time environments in computer graphics. A model specifically designed for realistic rendering of large-scale scenes is proposed by [34]. Stumpfel [35] is another researcher who worked on illumination of sun and sky to produce realistic environments.

Daylight is a combination of all direct and indirect lights originated from the sun and the diffuse of other objects. In other words, daylight includes direct sunlight, diffuse sky radiation and both of them reflected from the earth and terrestrial objects. Intensity of skylight or sky luminance is not uniform, awry and depends on the clarity of the sky [32].

The sun and sky are the main sources of natural illumination. The sun is a luminary that simulates the effect of sunlight and can be used to show how the shadows cast by a structure affect the surrounding area. The angle of the light from the sun is controlled by ones location, date and time. Sky light is most important outdoor illumination to make the scene realistic [2].

Hosek et al. [36] did a critical job on sky colour generation, based on Perez model which suffers from turbidity. Realistic sky colour is still based on [37] and [34] technique that we use as well.

To achieve a realistic mixed reality, shadows play an important role and are indispensable factors for 3D impressions of the scene [38] [39] [40]. AR simulation of shadows for a virtual object in real environments is difficult because of deeds reconstruction of the real-world scene, especially when details of approximation of the real scene geometry and the light source are known [41]. Jacobs et al. [42] prepared a classification of the illumination methods into two different groups, common illumination [41] [43] [44] [45] [15] [46] and relighting [47] [48] in mixed reality. The credibility of shadow construction with the correct estimation of light source position can be found in [48] [6] [49] [50].

Casting virtual shadows on other virtual and real objects is one of the existing issues in augmented reality. Haller et al. [45] modified shadow volumes to generate shadows in AR. In this algorithm a virtual object such as the real one but not more accurate is simulated which is called phantoms. The silhouette of both the virtual and the phantom objects are detected. Phantom shadows could be cast on virtual objects and virtual shadows could be cast on phantom objects. This method requires many phantoms to cover the real scene. Silhouette detection, the expensive part of shadow volumes is the main disadvantage of this technique especially when it comes complicated scenes. To recognize a real object as well as generation of the phantoms, is another problem with this algorithm.

Jacobs et al. [41] introduced a technique to create the virtual shadow of real objects with respect to a virtual light source where the real objects and the virtual light source are equipped with 3D sensors. Projection shadows are used for simpler objects while Shadow Maps (SMs) [51] are applied for more complicated ones. They proposed a real-time rendering method to simulate colour-consistent shadows of virtual objects in mixed reality.

Yeoh et al [18] proposed a technique for realistic shadows in mixed reality using a shadow segmentation approach which recovers geometrical information on multiple faded shadows. The paper focused on dynamic shadow detection in a dynamic scene for future requirements in mixed reality environments. The technique is similar to Shadow Catcher in [52] but in dynamic scenes.

Aittala [19] applied Convolution Shadow Maps [53] to produce soft shadows in AR which employed both mip-map filtering and fast summed area tables [54] to enhance blurring with variable radius. The method is applicable to both scenes external and the internal scenes.

Castro et al. [23] advised a method to produce soft shadows with less aliasing which uses a fixed distance relative to the marker, but with only one camera. The method also performs one sphere mapping such as [49], but selects a source or sources light most representative of the scene. This is important because of hardware limitations of mobile devices. The method supports self-shadowing as well as soft shadowing. They used filtering method such as Percentage Closer Filtering (PCF) [55] and Variance Shadow Maps (VSMs) [56] to generate soft shadows.

For the consideration of sunlight and skylight, [24] proposed an outdoor image by taking into account the sun and sky light with a linear combination as a basis image. The intensity of both sunlight and skylight are achieved by solving the system equation. This research could obtain the effect of environments on virtual objects in a fixed viewpoint. The main issue dealing with existing intrinsic image decomposition approaches is unreliability of natural captured image with a little control. Manually picking up some regions of the image to find a desirable sun and sky light, to make the algorithm reliable is another problem.

Knecht et al. [57] applied a technique in radiosity for blending the virtual objects into the real environments. Some shortcomings such as bleeding the light and double shadowing resulted in combining the instant radiosity and differential rendering [57]. The final work avoids inconsistent colour bleeding artifacts.

Kán et al. [58] used ray tracing method and applied photon mapping to enhance the realism of virtual objects as well as visual coherence between real and virtual objects in a composited image.

Madsen [9] estimated the outdoor illumination conditions in AR systems based on detecting the dynamic shadows. They used shadow volumes for generating virtual shadows. The direct sun and sky radiances from pixel values of dynamic shadows in live video are taken into account.

None real-time rendering is caused due to gathering many samples of the background image at different times which is the main difference with our approach in this study [59], [60]. Liu et al. [7] and Xing et al. [8] presented a static approach which could consider the outdoor illumination by taking advantage of essential association of the illumination factors and statistic attributes of each video frame. Such as previous work of this author, this research is viewpoint dependent. A desired future work of these researches was to obtain this results, but for real-time rendering which is our approach.

The biggest issue with augmented reality is the exact illumination with respect to the environments to make the system maximally realistic [16] [18] [19] [61] [62] [20] [63] [8] [9]. In the case of indoor rendering light colour and effect of other objects on virtual objects and vice versa is important which can be taken into account to make objects more realistic. In the case of outdoor rendering and involving sun and sky light the effect of skylight or sky colour plays a more significant role [64].

Kolivand et al. [65] proposed a technique to apply the effect of the sky colour on virtual objects in augmented reality in any specific location date and time. The main issue with the method is casting shadows on flat surfaces only due to the use of projection shadows for shadow generation. In this study we have tried to overcome the previous issue regarding to casting virtual shadows on other virtual and real objects with respect to the interaction between sky colour and augmented objects like what can be seen on real objects during daytime.

## Methods and Materials

### Sky Modelling

Before determining position of the sun, the sky must be modelled [66]. For creating the sky, virtual dome is a convenient tool. There are two ways to model the dome; using 3D modelling software such as 3D Max Studio, Rhino or Maya and using a mathematical function. Mathematical modelling is adopted for this real-time environment since it is easy to handle in the case of real-time. The dome is like a hemisphere in which the view point is located. Suppose that earth is a sphere. Julian date is a precise technique to calculate suns position [67]. The position could be calculated for a specific longitude, latitude, date and time using Julian date. The time of day is calculated using the Equation 1. 
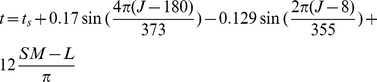
(1)where,


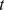
: Solar time


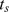
: Standard time


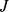
: Julian date


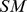
: Standard meridian


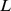
: Longitude

**Figure 2:**
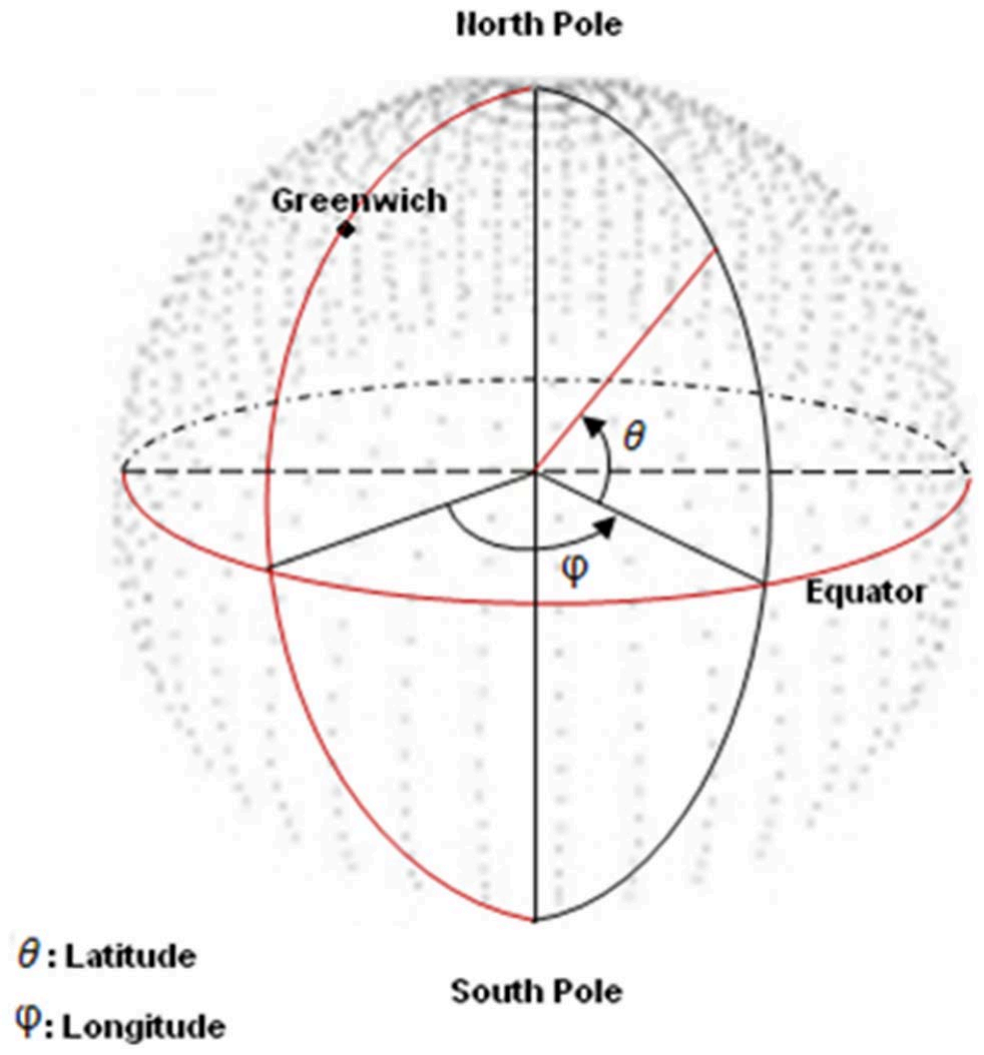
The zenithal and azimuthal angles on the hemisphere.

The solar declination is calculated as Equation 2. The time is calculated in decimal hours and degrees in radians. Finally, zenith and azimuth can be calculated as follows: ( Equation 3 and 4): 
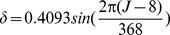
(2)

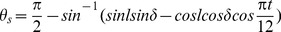
(3)

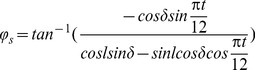
(4)where 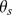 is solar zenith, 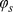 is solar azimuth and 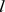 is latitude. With calculation of zenith and azimuth (Figure 2)suns position will become obvious.

### Perez Sky Model

The model is convenient to illuminate arbitrary point 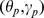 of the sky dome with respect to the position of the sun. It uses CIE [68] standard and could be used for a wide range of atmospheric conditions. Luminance of point 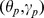 is calculated using the Equations 5 and 6: 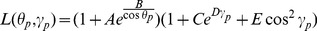
(5)

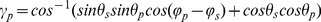
(6)


Where:

A: Darkening or brightening of the horizon

B: Luminance gradient near the horizon

C: Relative intensity of circumsolar region

D: Width of the circumsolar region

E: Relative backscattered light received at the earth surface

Essentially, to use Yxy space, the following three components are needed. In each point of view, the Y luminance is calculated by: 
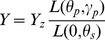
(7)


The chromaticity of x and y is calculated by: 
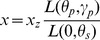
(8)

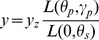
(9)


To colour each sky pixel, all of the pixels in the introduced formulae must be calculated iteratively. Involving date and time in specific locations enables the exact colour reproduction of each pixel.

### Z-Partitioning, Gaussian and Fogs Shadow Maps (Z-GaF Shadow Maps)

Shadow maps are convenient for casting shadows on other objects but suffer from aliasing. Applying Z-partitioning on conventional shadow maps and setting the resolution of the partitions could solve the aliasing out as many other works mentioned in the literature have. Semi-soft shadows are the most suited types of shadows which could be considered for outdoor rendering. To generate semi-soft shadows Gaussian approach is employed on the improved shadow maps using Z-partitioning. Although shadows demonstrate the actual distance between objects in virtual reality, AR systems still seem to lack the distance between real and virtual objects. Virtual objects usually appear nearer to the camera resulting augmented objects. In outdoor AR systems, this issue is met more than indoor rendering due to long distances and wide areas in outdoor environments. Applying a specific parameter of Fog [69] in the spacial partition of the view frustum which is split in advance, makes the virtual objects appear far from camera and consequently suitable for far distances in outdoor environments. The algorithm is summarised as shown in Algorithm S1.

**Figure 3:**
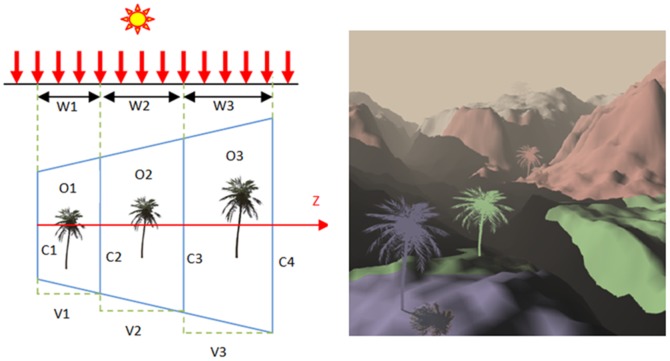
Left: theory of Z-GaF Shadow Maps when light and view direction are perpendicular, Right: Z-partitioning with 3 partitions in 1024*1024 resolution.

Applying Z-partitioning and Gaussian approximation on shadow maps reduces aliasing through increasing high resolution for areas in the scene that are closer to the point of view and decreasing the resolution for areas of the scene that are far away (Figure 3(Left)). Z-partitioning was done by splitting the camera view frustum into segments and filling the z-buffer for each segment separately(Figure 3(Right)). Assigning convenient resolution to each fragment depends on the fragment's z-value. This idea is used for wide scenes such as large terrain.

View frustum splitting is based on the earliest technique [70] and starts from the first object in the scene. This allows the GPU to be independent of the parts of the scene which are out of any rendering contribution. This, in addition to making the algorithm much faster, reduces the number of layers considerably.

**Figure 4:**
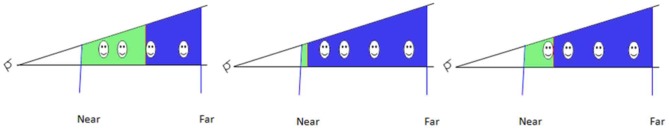
Split schemes, Left: Uniform splitting, Center: Logarithmic splitting, Right: Practical splitting.

View frustum splitting allows a shadow map to be generated and to change the resolution of each split part. The different types of splitting have an effect on the final quality and rendering speed. Uniform splitting, logarithmic and practical splitting schemes are the common types of splitting as can be seen in Figure 4.

Although parallel split schemes are proposed for reducing the aliasing, a uniform split scheme does not rectify the aliasing problem. The uniform distribution of perspective aliasing behaves no differently from standard shadow mapping. In this case, the perspective aliasing increases hyperbolically when the objects moves towards the camera. The logarithmic scheme is convenient for near objects but as objects are not located in front of the camera, it is not suitable in general cases.

**Figure 5:**
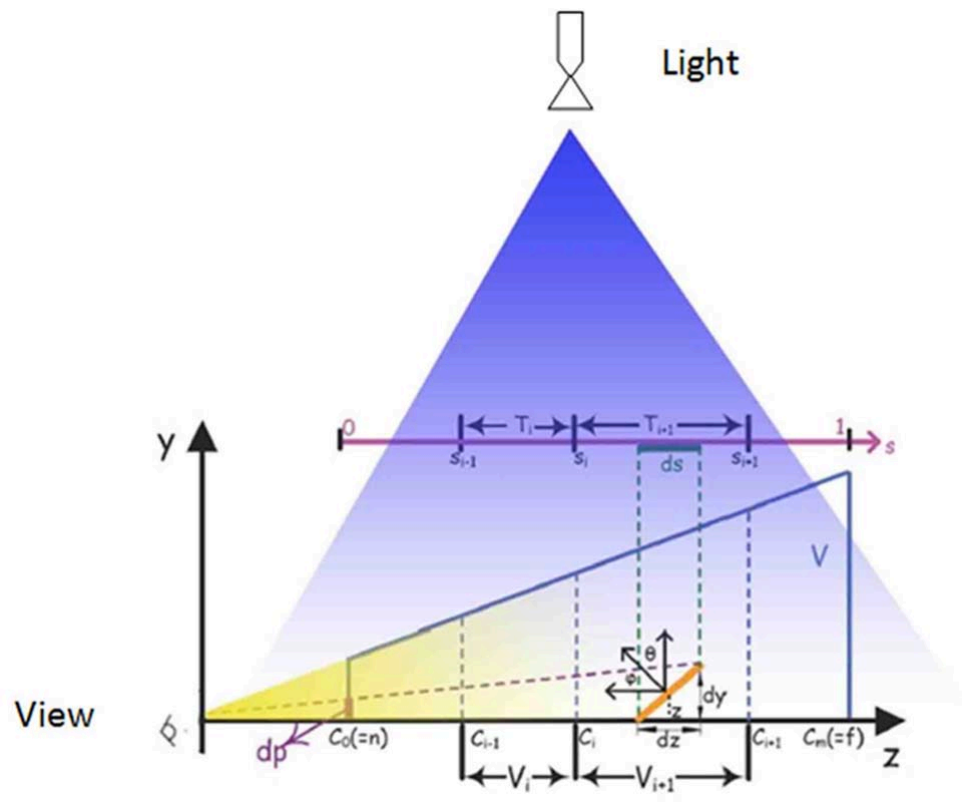
View frustum and light frustum mix to create Parallel Split Shadow Maps.

As logarithmic and uniform schemes could not cover the anti-aliasing for both near and far objects, taking their average could be beneficial (Figure 5). Simply put, if 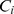 is a 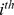 split of practical splitting, then 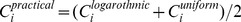
(10)


The presented technique for splitting is applicable for near and far objects. This technique requires non-negative bias to adjust the clip situation. There are some simple ways to reduce the bias. Increasing the precise depth is a method better suited to the near and far plane of the camera frustum.

Splitting whole scenes into multiple partitions helps control the resolution in different parts of a scene. A major difference between cascade shadow maps and the new approach is the non-uniform partitions.

In the proposed technique, there is no extra bias and it can be applied to bias concerns in most cases. A drawback of the proposed technique was evident when the light frustum was parallel to the view frustum.

Approximating the depth distribution using Gaussian approach, not only generates smoother shadow boundaries but also reduces the computational and storage cost.

The best way to create the illusion of depth is to take the colour value into account with respect to the distance from the viewpoint, which is fog employment. Fog is one of the widely used effects in most outdoor games whereby the size and the reality of the environments are realised. By enabling the depth testing and the fog, choosing the fog mode, fog colour, and fog density for the closest partition which is set by high resolution, realistic fog effect is constructed. The fog reduces the sharpness of the virtual objects. Therefore, far away virtual objects appear to fade into background similar to what can be seen in real environments. By setting the starting and ending distances for the fog not only in the first partition but also for any other partitions, fog can be applied on any specific virtual object.

In situations where the light direction is not perpendicular to the view direction (Figure 4(Left)), splitting the depth map into non-intersection layers and creating one shadow map for each layer in the light space could cover the redundancy. Each shadow map is generated through irregular frustum clipping and scene organisation. This makes it possible to have different shadow maps without any intersection sample points. 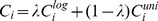
(11)

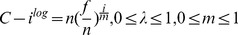
(12)

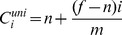
(13)


Where


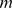
 is the number of splits, 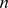 and 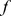 are near and far plane clippings, respectively. 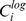 and 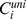 are two classic splitting schemes that increase details by referring to [71]. 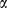 is the split position weight which depends on the practical requirements of the application.
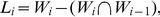
(14)


Where 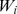 is light frustum splitting with respect to view frustum splitting (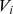) and 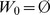.

## Implementation and Results

### Z-GaF Shadow Maps

**Figure 6:**
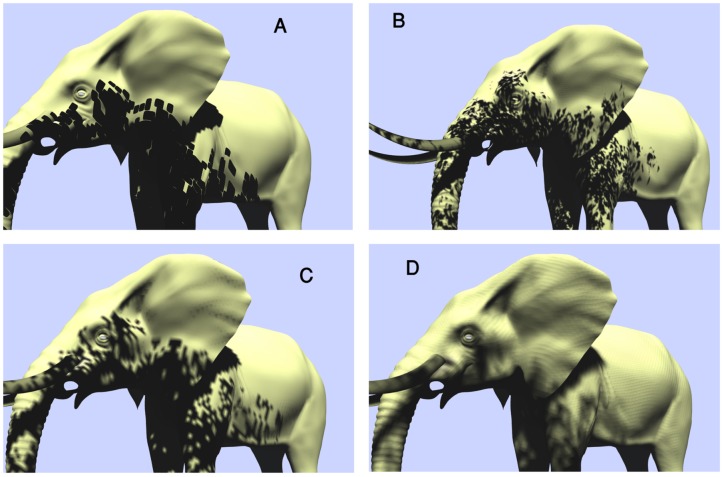
(A): The first two steps of Z-GaF which is conventional Shadow Maps, (B): Applying the presented Z-partitioning, (C): Applying Gaussian approximation on shadow maps, (D): Soft Shadows.

Implementing the two first steps of Z-GaF algorithm are the conventional shadow maps whose results are illustrated in Figure 6 (A). All pictures are captured in 1024*1024 resolution. The shadows of the tree are cast on the elephant. Self-shadowing can be observed on some parts of the elephant's body especially shadows of the ears and ivories. In Figure 6 A aliasing is the main issue which made the shadows less realistic.

**Figure 7:**
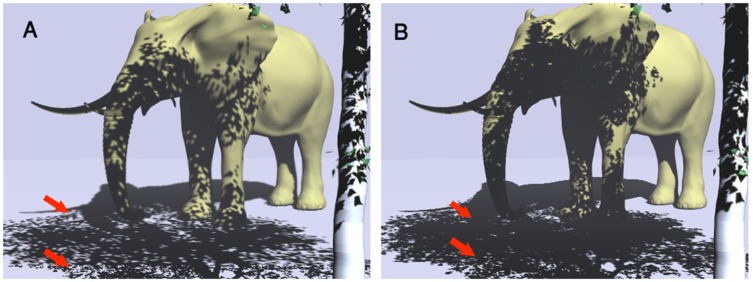
(A): Logarithm splitting, (B): Practical splitting.

Splitting the Z-depth to 2 to 4 partitions depends on the distance of the objects from the cameras viewpoint allows to change the resolution of each partition (Figure 6 B). High resolution generates high quality while producing low FPS. The close partitions are set with high enough resolution to enhance the realism of objects. Low resolution reduces the time of rendering, consequently increasing the speed of rendering (Figure 6C and (D)). Obviously, when a wide scene like an outdoor environment is rendered with the same resolution, some parts of it which are located far away from the camera, may not be seen very well, wasting the GPU's and CPU's time. Therefore, they are performed in low resolution. Practical splitting is tested to generate appropriate distribution of the partitions which can be seen in Figure 7.

Higher resolution results in a higher shadow quality, but suffers from an increase in rendering time. To overcome this problem and keep the trade-off balance between quality and rendering speed, the view frustum is split into different partitions. The number of partitions can be set manually. Figure 7 shows the difference between our proposed technique and previous ones for determining the best suited splitting. To enhance the quality of shadows the close partitions are set with a higher resolution, while in case of reducing the time of rendering, far partitions are set with a lower resolution. Results of assigning low resolution on some of the partitions can be observed in Table 1 in case of high enough FPS.

**Table 1:** Speed of rendering measured by FPS in different resolutions.

Method	1024*1024	2048*2048
SMs	122	116
PCF	75	63
CSMs	84	79
Z-GaF	96	90

In Figure 7 (A) the partition distribution is based on logarithm function. The partition's location is not appropriately selected. In Figure 7 (B) partitioning is constructed based on Practical splitting. The beginning of each partition is marked by a red arrow.

**Figure 8:**
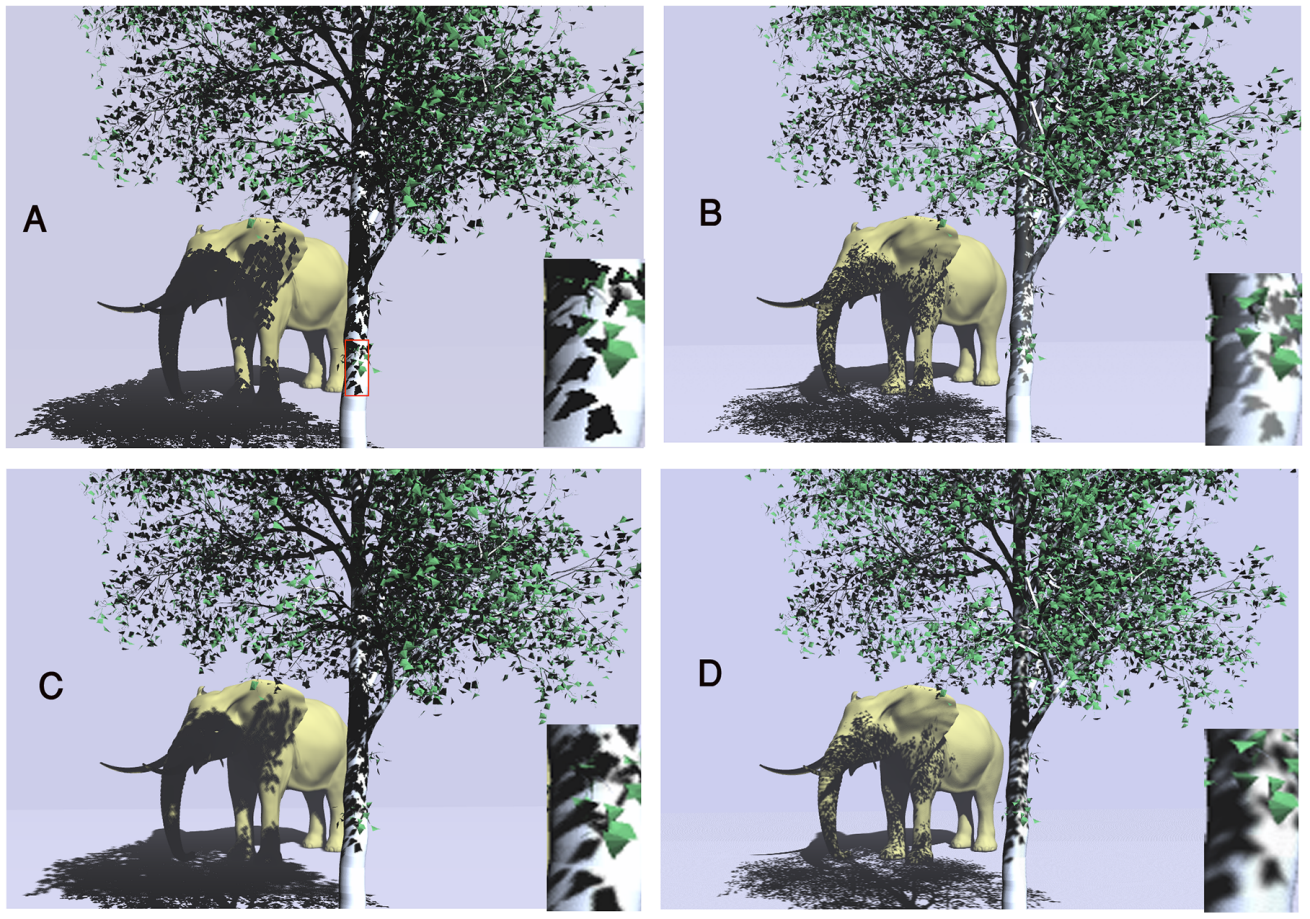
(A): Shadow Maps, (B): PCF, (C): CSMs with Gaussian blurring, (D): Z-GaF Shadow Maps.

Integration of the presented approach for Z-partitioning and Gaussian approximation not only generates a convenient semi-soft shadow compared to PCF and Cascade Shadow Maps (CSMs) [72] but also, there is no light leaking as compared with VSMs [56] and Layer Variance Shadow Maps (LVSMs) [73]. The main concern of VSMs and Convolution Shadow Maps (CoSMs) [71] is light bleeding due to Chebyshev Inequality for the upper bound of light visibility test and exponential approximation, respectively. Our upper bound approximation, which is based on Gaussian distribution for all layers generates a semi-soft shadow or somehow soft shadows as can be seen in Figure 8 (D).

Figure 8 draws a comparison between previous algorithms and Z-GaF Shadow Maps in 1024*1024 resolutions. Figure 8 (A) is the result of conventional Shadow Maps while (B) is the result of PCF. Figure 8 (C) illustrates CSMs using Gaussian blurring. Figure 8 (D) is the result of Z-GaF Shadow Maps which is an accurate shadow with semi-soft shadows for outdoor environments.

### Integration of Sky colour and Z-GaF Shadow Maps

Integration of sky colours and Z-GaF shadow Maps in real-time environments is performed successfully. Z-GaF Shadow Maps could produce high quality semi-soft shadows compared to previous algorithms. By combination of Z-GaF Shadow Maps and sky colour using a friendly GUI to set-up the specific location, date and time, an outdoor rendering application is provided. In the next section an evaluation on proposed integration is presented in details.

**Figure 9:**
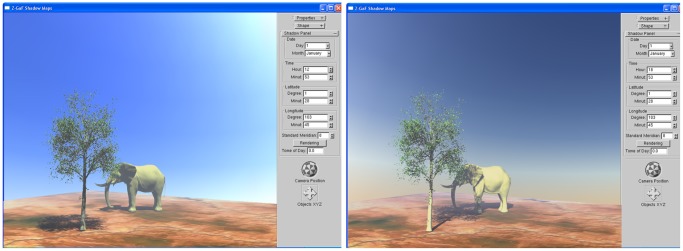
Integration of Z-GaF Shadow Maps and sky colour, January 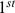 at Universiti Teknologi Malaysia at different times of day.

Figure 9 illustrates the implementation of Z-GaF Shadow Maps and sky colour. The effect of sky colour can be observed from the pictures.

**Figure 10:**
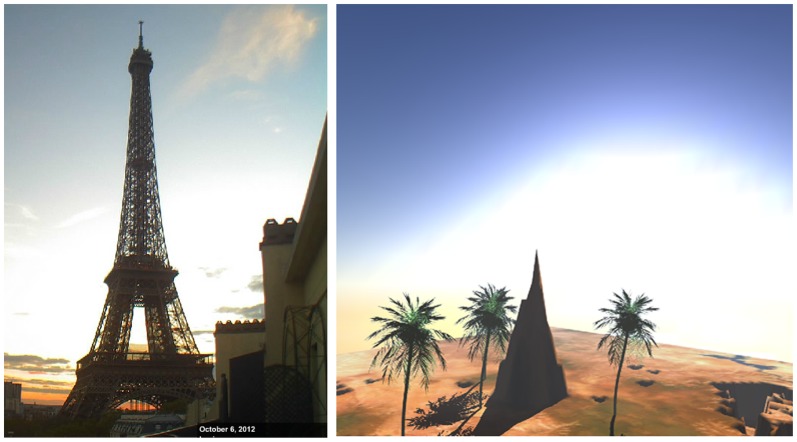
Left: Eiffel Tower, captured on 6 October at 16∶03 (Source: http://www.earthcam.com/), Right: The Software generated result for Eiffel Tower position on 6 October at 16∶03 (http://www.flickr.com/photos/118766222@N04/12784543685/).

**Figure 11:**
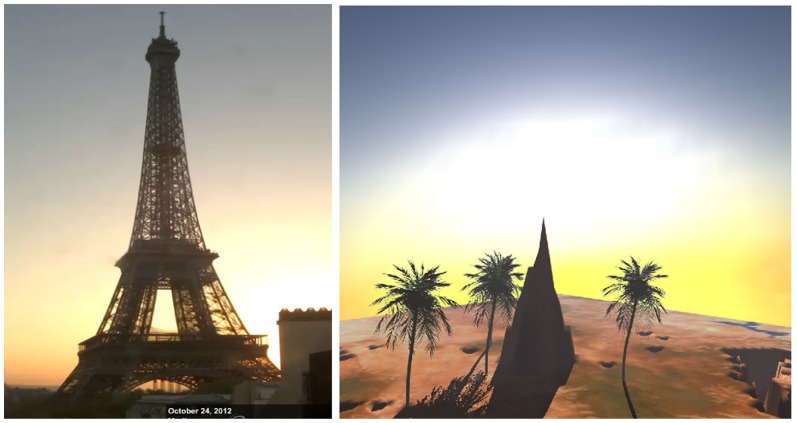
Left: Eiffel Tower, captured on 24 October at 16∶23 (Source: http://www.earthcam.com/), Right: The Software generated result for Eiffel Tower position on 24 October at 16∶23 (http://www.flickr.com/photos/118766222@N04/12784631475/).

**Figure 12:**
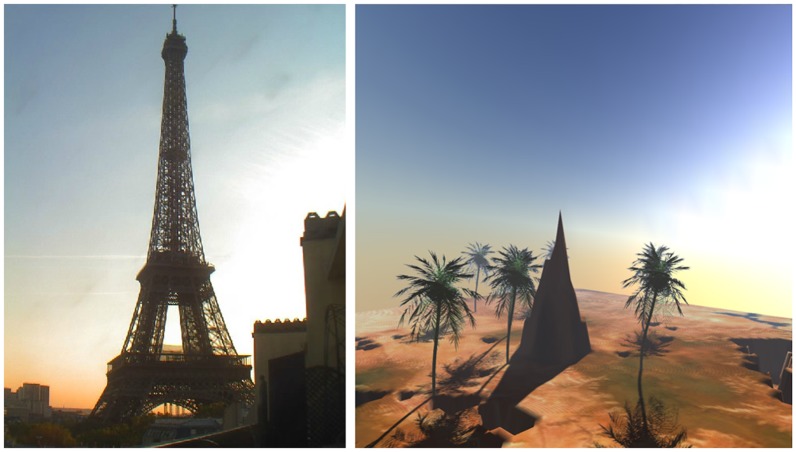
Left: Eiffel Tower, captured on 5 September at 17∶19 (Source: http://www.earthcam.com/), Right: The Software generated result for Eiffel Tower position on 6 September at 17∶19 (http://www.flickr.com/photos/118766222@N04/12785060384/).

There is a majority of web camera service providers on the World Wide Web, however, most of the web cameras do not grant a view of the sky. Many of them show traffic or crowds. Only a few web cameras capture the sky panorama. Figures 10, 11 and 12 shows some images of the Eiffel Tower in Paris, captured from France-Telecom (2012) at different points in time, in different days.

## Discussion

Extra stages are not needed to generate virtual shadows on virtual objects through implementing Z-GaF Shadow Maps. Since they are based on shadow maps, casting the virtual shadows on other objects is the main ability of this category of shadow generating techniques.

**Figure 13:**
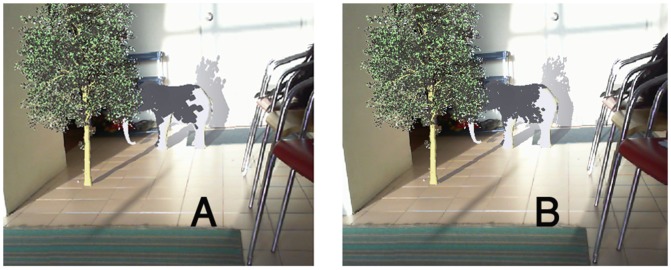
(A) Conventional Shadow Maps on virtual and real objects, (B) PCF shadows on virtual and real objects.

Figure 13(A) illustrates a scene including two virtual objects, a tree and an elephant. The virtual shadows of the tree are cast on the virtual elephant and the real wall simultaneously. The shadow technique used in the left picture is that of simple shadow maps with 512*512 resolution which does not produce any adequate results. Applying PCF with 1024*1024 resolution in the right side picture yields better results ( Figure 13(B)).

**Figure 14:**
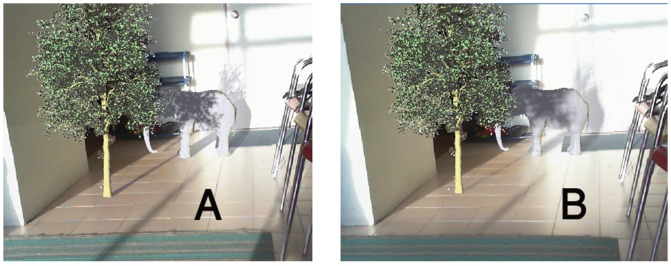
Casting Z-GaF Shadow Maps on virtual and real environments simultaneously, (A) Z-GaF Shadow Maps, (B) Soft shadows using Z-GaF Shadow Maps.

Figure 14 is the exact scene which was presented in Figure 13. In these pictures Z-GaF Shadow Maps are applied instead. In picture (A), Z-GaF Shadow Maps without blurring cast virtual shadows on real and virtual objects, while in the picture (B), Z-GaF Shadow Maps and Gaussian approximation is employed to generate soft shadows.

**Figure 15:**
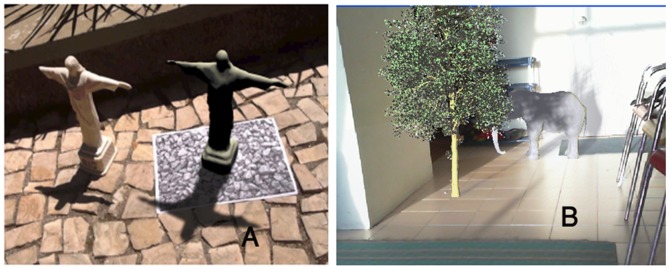
(A) Castro results [23], (B) Our results.

Castro et al. [23] proposed a method to produce semi-soft shadows with less aliasing using a fixed distance relative to the marker, but with only one camera (Figure 15 (A)). The method also performs one sphere mapping such as [49], but selects a source or sources of light, most protruding at the scene. This is important because of hardware limitations of mobile devices. The method does not support self-shadowing and soft shadowing. They used filtering methods such as PCF ([55] and VSMs ([56] to generate soft shadows. To compare our research with [23], Z-GaF Shadow Maps is implemented to cast soft shadows on other virtual and real objects includes 10 million triangles which can be seen in Figure 15 (B).

Collectively, Z-GaF Shadow Maps could be performed in augmented reality environments to generate shadows on other objects without any aliasing and light bleeding. They are also suitable to be applied for soft shadow generation not only in virtual environments but also in augmented reality systems.

### Interaction Between Sky colour and Object in Outdoor AR Environments

The technique integrates position of the sun, sky colours, shadows and interaction between sky colours and augmented objects. Position of the sun is managed, using Julian date and a GUI for setting the location, date and time. The sky colours are generated based on Perez model [37] but are not visible for the camera as the dome is beyond the view of frustum. Augmented objects are uploaded as OBJ models. Both marker and markerless techniques are applied to control the location, direction and orientation of the augmented objects. Shadows are appeared using Z-GaF Shadow Maps. The generated sky colour exerts the energy of each patch to the all visible patches of augmented objects using RCC [65]. Convergence rates could be set through the GUI to find out the best interaction compared to the real objects. The desired interaction is achieved by comparing to the real objects which are the best benchmark for the current work as the manner advocated by most researchers [74] [75] [24] [7] [17] [8].

The implementation during its first stage starts from ARToolkit with multiple markers loop function as the starting point and then calls a function to render an OpenGL GLUT scene, passing the geometry of the scene as function parameters. The GLUT scene function calls another GLUT display method in the OpenGL GLUT. The method calls the initializations of the scene, calls the display loop, and determines the geometry of the virtual scene. Knowing that the shadows, depending on Z-GaF Shadow Maps, of each object are rendered within the scene itself, it would be much easier for a programmer to render the shadows in AR environments. Moreover, to show more realistic interaction between augmented and real objects a similar looking-like primitive alpha objects for the background of the virtual environments is taken into consideration to cast the shadows on real environments.

For the AR environment Z-GaF Shadow Maps are employed. The sky colour is constructed but remains invisible. The 3D objects are loaded using a simple markerless technique in a wide scene. Position of the sun is traced by setting the location, date and time. The effects of generated sky colours are applied on the virtual objects during the day using Algorithm S2. The position of the viewer is set by changing the position of the system or set according to the real suns' current position. By employing these techniques following results are obtained:

Where, 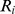, 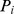, 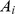 are radiosity, reflection, and area of patch 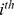 respectively. 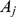 is the area of patch 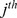, and 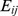 is the amount of energy from 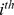 patch to 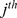 patch. 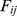 is form factor from 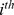 patch to 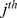 patch [76]. Implementation of RCC is employed successfully and a video of the results is posted in youtube (*http://www.youtube.com/watch?v=RHbb0fgpw8Y*).

Regarding the revelation of the interactions between sky colours and virtual objects in AR, wide surfaces for virtual objects are convenient compared to thinner ones. Trees have been selected to make the scene more complex and elephants because of their wide enough skin to show the amount of sky colour energy absorption on the virtual objects.

**Figure 16:**
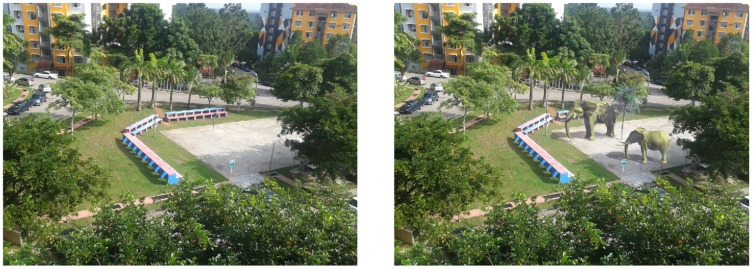
A scene with and without augmented objects, at 9∶55 on January 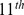 2013 at Universiti Teknologi Malaysia.

In a wide outdoor AR scene, markerless technique is performed to make the environments more realistic and indistinguishable than the real objects. Figure 16 (left) is a real scene, while (right) is the scene with three virtual objects and their shadows. Casting shadows on virtual objects(Big elephant) is one of the advantages of Z-GaF Shadow Maps which makes the system more realistic.

**Figure 17:**
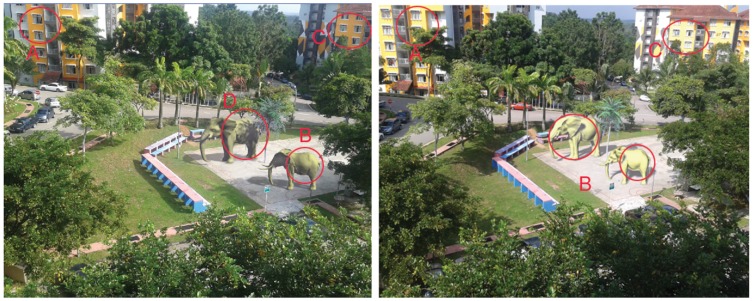
Interaction between sky colour and objects in augmented environment at different times of January 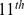 2013 at Universiti Teknologi Malaysia.

Figure 17 (left) is an augmented scene with three virtual objects (two elephants and a tree) which is captured at 9∶55 in January 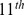 2013 at Teknologi University, Malaysia, where (right) is the scene with the three virtual objects which is captured at 15∶28, same day. The interaction between sky colours and objects is dstinct in these two pictures. The real interaction can be seen in the areas of 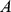 and 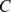 which are marked on the pictures. Area 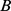 shows the virtual interaction. As compared with the 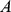 and 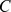 the results are accepted. The area 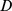 shows the shadows on other objects. In Figure 17 (left) when the real objects are darker due to the real sky colour, virtual objects also follow the effect of real sky colours using the proposed technique. In Figure 17 (right) when the real objects are lighter due to the real sky colour, virtual objects are also lighter.

**Figure 18:**
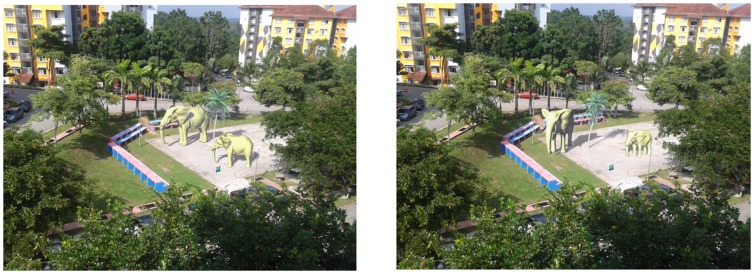
Rotating the virtual objects using mouse and keyboard in augmented environment at 15∶28 on January 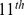 2013 at Universiti Teknologi Malaysia.

Figure 18 (left) is an augmented scene with the three virtual objects which was captured at 15∶28 in January 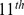 while (right) is the same scene in different orientation. When the location or orientation of augmented objects changes, the shadows remain in the same direction as the real ones.

The results posted in this section show the processes by which the objectives and consequently the aim of the research are achieved. The sky colours, shadows and the effects of the sky on virtual objects in the AR system are applied progressively.

## Conclusion and Future Works

This study provides a technique to demonstrate the interaction between sky colours and virtual objects in an augmented reality taking shadows into account. The main research contribution, in addition to shadow improvement, is the appearance of realistic virtual objects in outdoor rendering augmented reality environments. It involves 3D objects, sky colour effects and shadows which enhance the realism of the AR systems.

In the first part, the sky colours with respect to position of the sun in any specific location, date and time is successfully constructed. Specific longitude, latitude, date and time are the required parameters to calculate the exact position of the sun. The position is calculated based on Julian date and the sky colour is created based on Perez model. The sky colour is implemented based on Preetham's method [34] that is analytic model like actual atmosphere used in outdoor rendering.

Another contribution of this research is a new algorithm to create shadows with higher quality and higher frames per second, when compared to other algorithms such as Layer Variance Shadow Maps and Cascade Shadow Maps. Z-GaF Shadow Maps have been tested to vindicate an increase in the quality in a typical application.

The integrated prototype has been tested for performance. It carries out what the users expect. The strategy of testing the results of the technique have carried out. These include precise choice of the test data. The software has been produced for testing purposes during the research. It has helped to show that the calculations and software results are free from error. The results have been compared with the real world environment as well.

Interaction between virtual and real objects, beyond the interaction between sky colours and objects can largely enhance the realism. Much work needs to be done to induce the influences of real objects on virtual ones and vice versa. Radiosity and Ray-tracing are the suggested techniques when tasks such as this are performed. The radiosity technique is a more complicated process, requiring improvements to become fast enough to be applied in augmented reality environments as well as virtual environments.

This software, in addition to helping game makers generate outdoor games without worrying about shadows position and sky colours at different times of day and different day of year, also makes it possible for teachers of physics to teach about Earth orbits and the effect sun has on shadows.

## Supporting Information

Algorithm S1 **Z-GaF Shadow Maps.**
(PDF)

Algorithm S2 **Radiosity Caster Culling (RCC).**
(PDF)
