# Supplementary material for: Realistic Real-Time Outdoor Rendering in Augmented Reality
Source: PLoS One. 2014 Sep 30;9(9):e108334. doi: 10.1371/journal.pone.0108334 (PMC4182460; doi:10.1371/journal.pone.0108334)
Supplement: Algorithm S1 — Z-GaF Shadow Maps. (PDF) [file pone.0108334.s001.pdf]

Algorithm 1 Z-GaF Shadow Maps

Step 1: Render the whole scene from the point of light source and put it in the z-buffer (depth map)
Step 2: Render the whole scene again but from the camera point of view and transfer view point rendering to the light source coordinate system for each pixel in view point rendering (Step 1)
Step 3: Split the depth buffer to m partitions
Step 4: Set the high resolution for close partition and low resolution for far partitions
Step 5: Apply Gaussian approximation on depth map for all partitions
Step 6: Consider a Fog effect on the far partitions
