# Supplementary material for: Realistic Real-Time Outdoor Rendering in Augmented Reality
Source: PLoS One. 2014 Sep 30;9(9):e108334. doi: 10.1371/journal.pone.0108334 (PMC4182460; doi:10.1371/journal.pone.0108334)
Supplement: Algorithm S2 — Radiosity Caster Culling (RCC). (PDF) [file pone.0108334.s002.pdf]

Algorithm 1 Radiosity Caster Culling (RCC)

Step 1: For each patch i
Step 2: Set the hemicube, calculate form factor F_{ij};
Step 3: For each patch j apart from i
Step 4: If $A_j.visible$ =true
Step 5: $\Delta Rad = P_j \Delta R_i F_{ij} A_i / A_j$
Step 6: $\Delta R_j = \Delta R_j + \Delta Rad$
Step 7: $R_j = R_j + \Delta Rad$
End if
Next j
Next i
$\Delta R_i = 0$
